# Apology Number Two

**Published:** 1895-07

**Authors:** 


### HOMŒOPATHIC PHYSICIAN,

A MONTHLY JOURNAL OF

HOMŒOPATHIC MATERIA MEDICA AND CLINICAL MEDICINE.

"If our school ever gives up the strict inductive method of Hahnemann, we are lost, and deserve only to be mentioned as a caricature in the history of medicine."—CONSTANTINE HERING.

Vol. XV. JULY, 1895. No. 7.

### EDITORIAL.

APOLOGY NUMBER TWO.—The editor regrets that he must again disappoint his readers in the editorial for this month. The subject intended for this number is Arsenicum-album. Unfortunately the claims upon his time of patients who are desperately ill and need unremitting attention have compelled him to set aside every other consideration. Moreover, Arsenicum is a drug that requires much care in its preparation, and this it is not likely to get at present. Finally, even if it were ready for the printer, it would be impossible to find a place for it on account of the large amount of other material that requires presentation to our readers.

